# RTS,S malaria vaccine pilot studies: addressing the human realities in large-scale clinical trials

**DOI:** 10.1186/s13063-019-3391-7

**Published:** 2019-05-31

**Authors:** Machteld van den Berg, Bernhards Ogutu, Nelson K. Sewankambo, Nikola Biller-Andorno, Marcel Tanner

**Affiliations:** 1 Institute of Biomedical Ethics and History of Medicine, University of Zurich Winterthurerstrasse 30, 8006 Zurich, Switzerland; 2 Swiss Tropical and Public Health Institute, Socinstrasse 57, 4051 Basel, Switzerland; 3 University of Basel, Petersplatz 1, 4001 Basel, Switzerland; 4 Kenya Medical Research Institute (KEMRI), Mbagathi Road, Nairobi, Kenya; 5 CREATES, Strathmore University (SU), Madaraka Estate, Ole Sangale Road, Nairobi, Kenya; 6 College of Health Sciences, Makerere University, Upper Mulago Hill Road, Kampala, Uganda

**Keywords:** Malaria vaccine, Pilot studies, Low-resource settings, Community engagement, Ethics, Transnational clinical trials

## Abstract

A malaria vaccine as part of the integrated malaria control and elimination efforts will have a major impact on public health in sub-Sahara Africa. The first malaria vaccine, RTS,S, now enters pilot implementation in three African countries. These pilot implementation studies are being initiated in Kenya, Malawi, and Ghana to inform the broader roll-out recommendation. Based on the malaria vaccine clinical trial experiences, key ethical practices for effective clinical trial research in low-resource settings are described. For successful vaccine integration into malaria intervention programs, the relational dynamics between researchers and trial communities must be made explicit. Incorporating community values and returning to research practices that serve the intended benefactors are key strategies that address the human realities in large-scale clinical trials and pilot implementation, leading to positive public health outcomes.

## Introduction

In 2017 malaria was attributed to 435,000 deaths and nearly half the world’s population was identified to be at risk of infection. Malarial disease carries an immense public health burden, and those at the highest risk for malaria are children living in endemic regions; it is estimated that 266,000 children under 5 years of age died from infection in 2017 [1]. Since the year 2000, significant work has been done towards reducing the burden of malaria, with much progress made [2]. This success is largely owed to insecticide-treated bed nets (INTs), indoor residual spraying (IRS), and artemisinin-based combination therapy (ACTs), contributing to a 40% reduction in clinical disease and a 50% reduction in *Plasmodium falciparum* infection [2]. While these figures looked promising, they were still far short of the WHO target of 75% reduction in malaria burden by the year 2015 [3]. In order to achieve this reduction, more tools are needed, including effective vaccines, especially in high-transmission areas.

Currently, a number of vaccines are in the pipeline for malaria, in both pre-clinical and clinical development, targeting both children and pregnant women [4]. These target varying stages of the malaria parasite’s life-cycle and are categorized as pre-erythrocytic vaccines (PEV), blood-stage vaccines (BSV), transmission-blocking vaccines (MSTBV), and combination vaccines. Despite a robust pipeline, long-lasting sterile immunity against malaria with these vaccines is unlikely and a vaccine will instead serve as a tool to be utilized in combination with other intervention methods. Due to the high burden of disease, a vaccine with a modest level of protection would translate into a significant impact given almost half a million annual deaths. Today there is cautious optimism for a modestly effective vaccine called RTS,S developed in partnership by PATH Malaria Vaccine Initiate (MVI), GlaxoSmithKline (GSK), and a number of academic and research institutions.

RST,S is a pre-erythrocytic vaccine that has been in development since 1987 and concluded phase III testing in early 2014. The phase III studies adhered stringently to all aspects of formal research requirements, such as Good Clinical Practice (GCP) and Good Laboratory Practice (GLP) guidelines; every effort was made to standardize the process. This large multi-national trial enrolled 15,459 infants and was carried out at 11 clinical trials centers in seven countries (Burkina Faso, Gabon, Malawi, Mozambique, Ghana, Tanzania, Kenya). RTS,S has been shown to be most effective in children aged 5–17 months, who receive three doses of the vaccine and then a booster at 20 months of age, reducing the cases of severe malaria by 36% [5].

The next step for this vaccine is pilot study roll-out in Kenya, Ghana, and Malawi. In these three selected countries the Ministries of Health will work together with the WHO to establish the Malaria Vaccine Implementation Program (MVIP). In addition to the efficacy and safety profile, these pilot implementation studies will shed light on the programmatic feasibility of RTS,S in real-life settings, a factor modeling studies have identified to be a critical component of reaching a significant public health impact [6, 7]. To adequately assess the feasibility of RTS,S in real-life settings, researchers must engage local communities and carefully consider the ethics and processes of undertaking MVIP in different sociocultural settings. This means acknowledging relational ethics in vaccine studies, community dynamics, and vaccine uptake and a commitment to long-term monitoring of the malaria vaccine program.

### The role of relationship in research studies

Previous RTS,S studies have placed a lot of emphasis on adhering to regulatory requirements and formal research ethics. What we can learn from other vaccine trials is that the emphasis of formal research ethics is as important as close engagement of the clinical trial team with the community [8]. Taking the time to build intimate relationships fostering shared ownership of the research synergistically and effectively complements the scientific protocols and formal ethical procedures. Relational ethics and shared ownership play a significant role in determining the effectiveness of malaria vaccine studies. Much of the success of a malaria vaccine trial in The Gambia has been attributed to intimate kinship-like relationships between the trial community and field workers [8]. This kind of a relational interaction fosters close collaboration between researchers and communities to confront social and political circumstances. Through this interaction it was found that even in contexts where parental awareness of the RTS,S vaccine was low, there remained a keen desire to enrol children in RTS,S vaccination programs if made available. Based on this work, recommendations have been made around disseminating information specifically to mothers about the vaccine that considers the social and political realities that participants inhabit [9]. In Tanzania, stakeholders have expressed a positive opinion of the vaccine but have drawn attention to the need for an inclusive communication strategy that incorporates communities and local health care professionals in a culturally appropriate way for that positive opinion to endure [10]. These examples shed light on the need to critically assess processes and ethical conduct during large-scale clinical trials to ensure effective incorporation of the tool into integrated malaria control and elimination programs, considerations that stretch beyond a confident safety profile.

The international community has set ambitious goals for malaria by 2030 and has put RTS,S on the table as a tool to be used in an integrated approach with other malaria interventions [11]. As we move forward these relational aspects are of particular importance to foster a concerted effort across scientific disciplines and stakeholders from varying sociocultural backgrounds. By harnessing lessons learned from previous malaria vaccine clinical trial experiences, effective integration of this vaccine into malaria control programs is possible in richly diverse sociocultural contexts.

### Communities and vaccine uptake

Strong community relations and local engagement have the potential to bring autonomy, transparency, and respect to the work of the researchers. From a utilitarian perspective, it can strengthen the RTS,S malaria pilot study in the selected sites. The RTS,S phase III studies had Community Advisory Boards (CABS) which consisted of influential community members who supported the communication between communities and researchers. These have been effective and therefore need to be further strengthened to involve additional stakeholders with interests in the community and the children’s welfare at large [12]. Acknowledging fundamental intelligence within communities and translating this into the execution of these pilot implementation studies will propel us toward the goal of RTS,S informed choice and acceptance within the community setting. Listening to the voices of the community and integrating these into the study design leads to greater research vaccine uptake [13–15]. Failure to do so heightens the power disparity and reduces optimal integration.

### Long-term monitoring of study sites

In malaria endemic regions, the risk of mortality shifts to morbidity at around age five. However, modeling studies suggest that severe malaria is likely to occur at later ages in children living in RTS,S research sites [6]. This places an emphasis on the need for long-term monitoring and evaluation of research sites. This sustained long-term monitoring is often resource consuming but is critical in understanding the impact of interventions and shifting disease epidemiology. Through the collection of narratives and genuinely engaging with community members to share their experiences, potential risks can be followed-up, flagged, and clinically investigated. Ethical conduct does not only conclude at obtaining informed consent, it continues into research, monitoring, and implementation. The transition in age for severe malaria needs to be explained to the communities—that it is not related to the vaccine deployment but rather a change in the disease prevalence. This, if not explained well, may affect the uptake of the vaccine over time.

Some may challenge the feasibility of relational engagement that is context-sensitive to the MVIP sites; however, substantive evidence indicates that close community engagement is a favorable approach to the research in question and does not introduce biases and dependencies of concern at the ethical or research outcome levels [16–19]. We therefore argue that this can be streamlined and, on the contrary, strengthen the efficiency of the pilot studies. Communities can always provide key insights and facilitate researcher familiarization with the research setting. This, in turn, allows for effective planning and site organization, two factors that have been identified to be key contributors to streamlining the efficiency of clinical trials [20]. Through encouraging dialogue during initial introductions and building relationships that encourage communities to speak up, to feel safe when doing so, and report when adverse events occur, time will be saved and researchers can ensure a more benevolent process as the monitoring is intrinsic to the study. Previous studies looking at the benefits of community engagement support these claims, suggesting enhanced ownership of the research by local communities strengthens the effectiveness of the research [21–24]. Combating the thorny dynamics of malarial disease without this approach can lead to the aggravation of adverse events and mistrust in the community of vaccination research as a whole [21, 24]. Therefore, it is the responsibility of those who will conduct the pilot implementation studies to call upon the communities to work alongside them for support when incorporating the distinctive values and social and cultural practices to build trust and ultimately strengthen the social value of RTS,S.

## Conclusions

Reaching coverage across the target population and creating an integrated, tailored approach alongside other malaria interventions will determine the extent of the public health impact a malaria vaccine will have [6, 25]. This means a keen awareness of local prevention and treatment practices as well as transmission patterns. Vaccines in the development pipeline targeting parasitic diseases cannot be compared to the well-known highly efficacious vaccines of the childhood diseases caused by bacteria and viruses. Parasites have very complex life cycles with sexual and asexual development cycles in different niches of the hosts, and thus current efforts lead to only partially efficacious vaccines that still show a significant alleviation of the disease burden [26]. Consequently, working with partially effective vaccines, and tools in general, heightens the emphasis we must place on rooting research in the local social realities to best understand compliance and adherence. RTS,S has an excellent opportunity to set the global stage for shared research ownership, effective community engagement, and the development of an integrated, tailored approach to reduce the disease burden.
